# Progress towards a more predictive model for hohlraum radiation drive and symmetry

**DOI:** 10.1063/1.4982693

**Published:** 2017-05-19

**Authors:** O. S. Jones, L. J. Suter, H. A. Scott, M. A. Barrios, W. A. Farmer, S. B. Hansen, D. A. Liedahl, C. W. Mauche, A. S. Moore, M. D. Rosen, J. D. Salmonson, D. J. Strozzi, C. A. Thomas, D. P. Turnbull

**Affiliations:** 1 Lawrence Livermore National Laboratory, Livermore, California 94551, USA; 2 Sandia National Laboratory, Albuquerque, New Mexico 87185, USA; 3 Laboratory for Laser Energetics, Rochester, New York 14623, USA

## Abstract

For several years, we have been calculating the radiation drive in laser-heated gold hohlraums using flux-limited heat transport with a limiter of 0.15, tabulated values of local thermodynamic equilibrium gold opacity, and an approximate model for not in a local thermodynamic equilibrium (NLTE) gold emissivity (DCA_2010). This model has been successful in predicting the radiation drive in vacuum hohlraums, but for gas-filled hohlraums used to drive capsule implosions, the model consistently predicts too much drive and capsule bang times earlier than measured. In this work, we introduce a new model that brings the calculated bang time into better agreement with the measured bang time. The new model employs (1) a numerical grid that is fully converged in space, energy, and time, (2) a modified approximate NLTE model that includes more physics and is in better agreement with more detailed offline emissivity models, and (3) a reduced flux limiter value of 0.03. We applied this model to gas-filled hohlraum experiments using high density carbon and plastic ablator capsules that had hohlraum He fill gas densities ranging from 0.06 to 1.6 mg/cc and hohlraum diameters of 5.75 or 6.72 mm. The new model predicts bang times to within ±100 ps for most experiments with low to intermediate fill densities (up to 0.85 mg/cc). This model predicts higher temperatures in the plasma than the old model and also predicts that at higher gas fill densities, a significant amount of inner beam laser energy escapes the hohlraum through the opposite laser entrance hole.

## I. INTRODUCTION

At the National Ignition Facility (NIF),^1^ experiments are being done to study the feasibility of fusion ignition using indirect drive.^2^ In this technique, lasers are used to heat a gold or an uranium cylindrical cavity, called a hohlraum, producing an x-ray drive that is used to implode a capsule. The capsule consists of a deuterium-tritium fuel layer surrounded by an ablator material (usually plastic or high density carbon (HDC)).^3^ The hohlraum has laser entrance holes (LEHs) at either end. The beams are pointed such that they illuminate the inside of the hohlraum in three rings of approximately equal energy—one ring at each end near the LEHs (“outer beams” entering the hohlraum at 44.5° or 50°) and one at the midplane (“inner beams” entering the hohlraum at 23.5° or 30°). For 15–20 ns long laser pulses required to implode a plastic capsule, a major challenge is to maintain radiation drive symmetry throughout the pulse by controlling the relative brightness of the laser illuminated rings. Late in time, the hohlraum begins to fill with a highly absorbing high-Z material ablated off the walls that attenuates the beams pointed at the midplane, generating an asymmetric radiation drive. One way to maintain late time symmetry control is to fill the hohlraum with a low-Z gas in order to hold back the ablated wall material and maintain a low-Z heated channel for the beams to propagate to the hohlraum midplane.

**FIG. 1.**
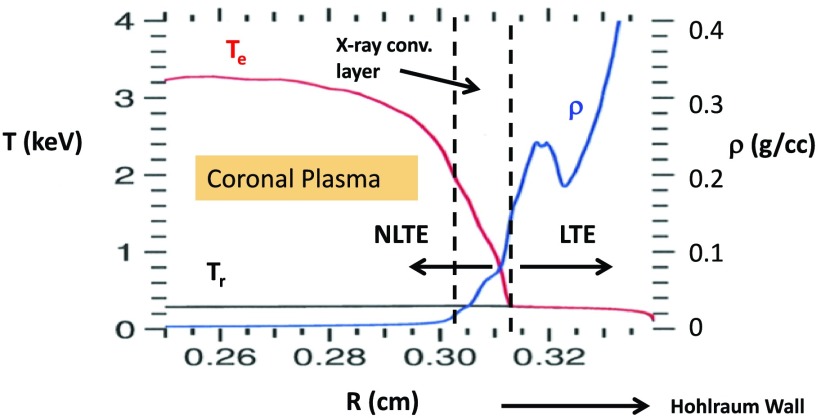
Radial lineouts of temperature and density at the z location where outer beams are depositing their energy from a calculation of a NIF 6.72 mm diameter laser-heated hohlraum.

**FIG. 2.**
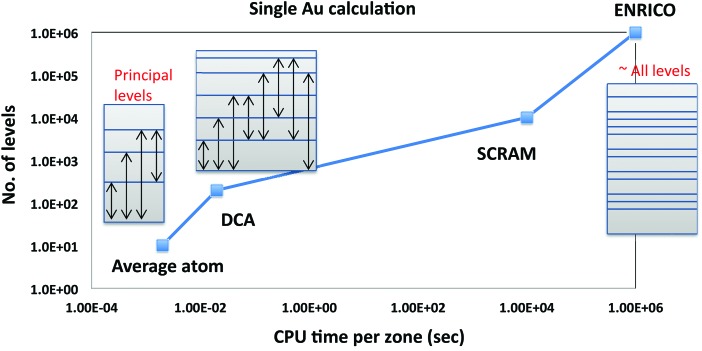
Approximate CPU time required to compute a single set of NLTE populations as a function of the complexity of the model.

We simulate laser-heated gas-filled hohlraums using the radiation hydrodynamic codes Lasnex^4^ and Hydra.^5^ Figure 1 shows a radial lineout at peak power of the electron temperature, T_e_, the radiation temperature, T_r_, and the density, ρ, at the location in z, where the outer cone lasers are being absorbed for a typical calculation of a laser-heated hohlraum. The laser heats and ablates the gold wall, which blows down to low density and forms a coronal plasma, where T_e_ ≫ T_r_. In this region, the atomic populations are not simply described by a Saha equilibrium at T_e_ local thermodynamic equilibrium (LTE) but by a collisional radiative equilibrium that is not in a local thermodynamic equilibrium described by T_e_ (NLTE). As we move closer to the hohlraum wall (to the right in Figure 1), the density increases and T_e_ decreases, but T_e_ > T_r_ and the plasma remains NLTE. This ∼100 *μ*m thick layer, from R = 0.303 to R = 0.313 in Figure 1, is where all the net x-ray power is produced. Deeper into the high Z wall, the collision rates are high enough that the atomic populations are described by Saha equilibrium (LTE) and T_e_ = T_r_. The calculated radiation drive and symmetry depend primarily on three processes: (1) the absorption of the laser light by the hohlraum plasma (where T_e_ is ∼3 keV in Figure 1), (2) the conduction of that heat to the thin layer in the gold wall plasma, where heat is converted to x-rays (ρ ∼ 0.01–0.1 g/cc, T_e_ ∼ 1 keV, and T_e_ > T_r_), and (3) the confinement of that radiation by the high-Z (gold or uranium) wall (where T_e_ = T_r_). The laser absorption is sensitive to the plasma temperature, which depends on the electron thermal transport. The conversion of thermal energy to x-rays in the wall plasma also depends on the thermal transport of heat from the laser-heated plasma to deeper into the wall plasma and on the NLTE emissivity of the high-Z wall material. The confinement of the x-rays depends on the LTE opacity of the wall material.

For the past few years, our modeling of NIF hohlraums has used a recipe of physical models called the High Flux Model (HFM).^6^ In the HFM, we use tabular LTE opacities for gold or uranium for electron temperatures below 300 eV. The tables are made from extensive calculations using the super transition array (STA) method.^7^ The calculated opacities of gold and gold/uranium mixtures using STA are consistent with data from double hohlraum experiments on Omega.^8^ For electron temperatures above 300 eV, we assume NLTE conditions and calculate the emissivity and opacity using DCA.^9^ Here, DCA does not mean “detailed configuration accounting” but instead refers to both a computational package with the radiation hydrodynamics codes and a particular type of atomic model that utilizes highly averaged states and other approximations to make the computation time per zone tractable. The HFM uses particular DCA models for gold and uranium that we will refer to as DCA_2010. The HFM also uses flux-limited electron thermal transport, with a limiter of 0.15.

### A. High flux model applied to gas-filled hohlraums

Compared to earlier (prior to 2009) calculations that used the XSN average atom model^10^ for the high-Z wall emissivity and opacity and a flux limiter of 0.05, the HFM was a significant improvement. Experiments on the Omega laser where 60 beams uniformly illuminated a gold sphere^11^ showed that a flux limiter of 0.15 was able to improve the agreement with the measured x-ray conversion efficiency (fraction of absorbed laser energy converted to x-rays) compared to using a flux limiter of 0.05. The HFM also brought the predicted radiation drive for early NIF vacuum hohlraums into better agreement with measured values.^12^ Finally, the HFM predicted a cooler plasma in NIF gas-filled hohlraums relative to the average atom based calculations and brought the predicted stimulated Raman scattering (SRS) spectrum closer to the measured spectrum.^13^ However, subsequent NIF implosion experiments showed the HFM-calculated capsule bang times in gas-filled hohlraums to be consistently earlier than measured.^14^ Note that these experiments had capsules with Ge-doped or Si-doped CH ablators, a pulse duration of ∼20 ns, and hohlraum gas fills of 1–1.6 mg/cc He. The bang time discrepancy (experimental_bang_time–simulated_bang_time) varied from ∼500 to 800 ps and was consistent with the simulated drive being 20%–30% higher than the actual drive. To match the experimental capsule trajectories and bang times, ad hoc multipliers were applied to the input laser power in HFM calculations.^14^ Subsequent experiments using a special hohlraum having a 100% LEH and a thin capsule that became transparent to x-rays during the peak drive provided further evidence that the radiation drive seen by the capsule was in fact 20%–30% lower than that predicted in HFM calculations.^15^

More recent NIF experiments using HDC ablators and hohlraum He fill densities from 0.03 to 0.6 mg/cc are in better agreement with HFM calculations than the earlier CH ablator experiments, but a discrepancy of ∼300 ps or 10% in the peak drive persists.^16^ Experiments in which the hohlraum gas fill density was systematically varied up to 1.6 mg/cc showed that for 5.75 mm diameter hohlraums and 2 mm diameter HDC capsules, the bang time discrepancy increases for helium fills above 0.6 mg/cc,^17,18^ rising to 800 ps at a fill density of 1.6 mg/cc. We should note here that a bang time discrepancy persists with HDC implosions in spite of two factors that should make them simpler to model and understand than the CH implosions:

First, because the HDC is ∼3 times denser than the CH, the transit time of the shocks through the ablator is less, resulting in shorter laser pulses (6–9 ns). As a consequence of the shorter laser pulse, there is less need to use a high-density hohlraum gas fill to control the wall expansion for symmetry control. Second, with shorter laser pulses and lower fills, laser plasma interaction (LPI) processes such as backscatter and cross beam energy transfer (CBET)^19^ are no longer important. This is a significant simplification because LPI processes occur over very small spatial and temporal scales and are very difficult to calculate.

In this paper, we report a series of highly resolved axisymmetric calculations of hohlraum-driven implosions of gas-filled capsules using the Lasnex code. All but one of the capsules were 80 *μ*m thick shells made of HDC with an outer radius of ∼1080 *μ*m. We also simulated one Si-doped CH capsule that was 187 *μ*m thick with an outer radius of ∼1100 *μ*m. They were driven by a two-step radiation drive (see, e.g., Fig. 4). The hohlraum gas fill density and hohlraum size varied as shown in Table I. For all of these shots, the wavelength separation between the inner and outer cones (Δλ) was close to 0, and the amount of CBET was calculated to be small. In the calculations, the measured time-dependent backscatter was removed from the input laser power. We show that by making physically motivated modifications to the HFM, we can construct a model that predicts the radiation drive flux and capsule bang times for low fill, low LPI hohlraums driving HDC capsules without requiring any ad hoc adjustments to the input power. We further show that this model predicts decreased coupling of the laser power into the radiation drive as the hohlraum gas fill density is increased.

**TABLE I.** Parameters of the experiments^17,18^ that were simulated.

Experiment No.	Capsule	He fill (mg/cc)	Hohlraum diameter (mm)	Laser energy (kJ)	Backscatter (%)	Δλ (Α)
N140915-003-999	HDC	0.06	6.72	1461	1.8	0
N140701-002-999	HDC	0.6	6.72	1496	4.2	0
N150301-003-999	HDC	0.6	5.75	1079	7.4	0
N160424-001-999	CH	0.6	5.75	1066	5.3	0
N150506-002-999	HDC	0.85	5.75	941	4.0	0
N150426-001-999	HDC	1.1	5.75	952	6.6	0
N150228-002-999	HDC	1.6	5.75	846	7.2	0.6

As a first step in this study, we performed a grid convergence study to make sure that these calculations were fully converged. The details and methodology of the convergence study are described in Ref. 20. Briefly, we refined the radial grid spacing, the angular grid spacing, and the photon energy group bins until the time-dependent radiation flux around the capsule (averaged over the annular volume extending from 1.1 to 1.5 times the original capsule radius) was converged to 1%, and the P2 and P4 Legendre moments of the stagnated hot spot shape were converged to 1 *μ*m. To converge the radiation flux, we found that we needed 180 photon energy bins spanning 10 eV–100 keV and ∼300 radial zones in the 30 *μ*m hohlraum wall. For converged wall zoning, we set the innermost wall zone thickness to 40 A and then increased the thickness of each succeeding zone by the ratio of 1.03. The convergence was tested by varying the wall zone ratio. For example, previous calculations using 85 energy groups and a wall zone ratio of 1.1 were found to have deviations on the flux of up to ∼10% relative to fully converged calculations. To converge on the P2 moment of the hot spot shape, we found that we needed 1.25° angular zoning. The calculation was considered converged with respect to the shape when doubling the number of angular zones changed the hotspot P2 by less than 1 *μ*m. Some previous calculations with 2.5° angular zoning had deviations in the P2 of ∼8 *μ*m relative to the fully converged result. Note that the main effect of the increased angular zoning is that it improves the resolution of the absorption of the inner beam light as it propagates through the hohlraum fill gas and ablated wall material.

## II. IMPROVED DCA NLTE MODEL

Recall that the net x-ray production in a hohlraum occurs in a thin NLTE layer just inside the LTE part of the wall. In simulated hohlraums, a NLTE emissivity (x-ray production) is calculated “inline” in each zone at each time step. To be practical in a hydrodynamic calculation involving many thousands of zones, it has to be done in fractions of a second. Figure 2 shows the approximate time to compute a single NLTE emissivity for gold as a function of the number of atomic levels included in the calculation. Highly averaged models such as the average atom or the DCA model are fast enough to be practical to run inline but sacrifice accuracy due to limited states and averaged structure and rates. More detailed atomic physics codes such as SCRAM^21^ and ENRICO^22^ take much longer to run but give more accurate answers.

**FIG. 3.**
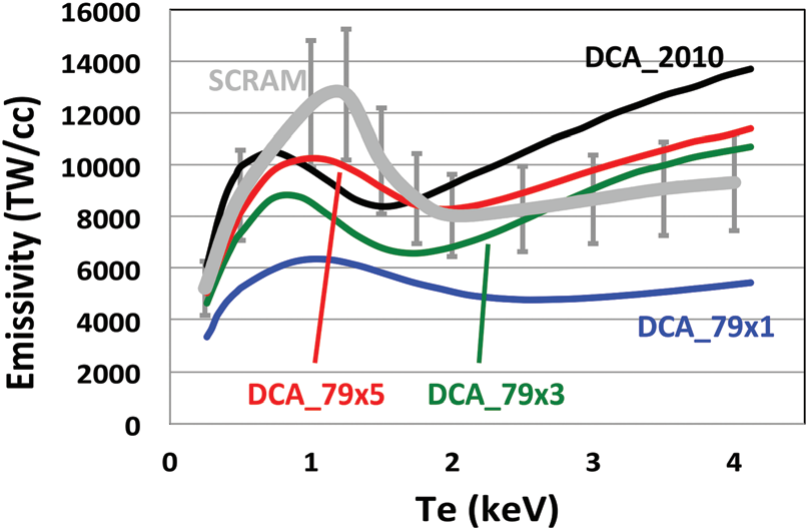
Plot of the spectrally integrated emission of Au at 10 mg/cc vs. T for detailed SCRAM calculation with 20% uncertainty bars compared to 4 calculations with DCA models: DCA_2010 (black), DCA_79x1 (blue), DCA_79x3 (green), and DCA_79x5 (red).

To assess the accuracy of the baseline DCA_2010 NLTE model, we compared it to a detailed offline SCRAM calculation by computing the spectrally integrated emissivity of 10 mg/cc gold at temperatures ranging from 0.5 to 4 keV. These calculations did not include a radiation field, and so, only collisional processes determined the atomic populations and emissivities. We then compared the DCA_2010 emissivity to the emissivity from 3 different DCA models and to the detailed SCRAM calculation. Figure 3 shows the emissivity versus T for several different NLTE models. The thick gray curve with 20% uncertainty bars is the most detailed calculation using SCRAM. This calculation requires ∼15 CPU minutes to evaluate a single emissivity and is therefore too slow to use in a hydrodynamic calculation. Comparing the SCRAM result to the baseline DCA_2010 model (black curve), we see that the DCA_2010 model is not emissive enough at 1 keV and too emissive above 2 keV as compared to SCRAM. Given these discrepancies, we tried 3 increasingly more detailed (but still highly averaged and computationally efficient) DCA models also shown in Figure 3. DCA_79x1 started from DCA_2010 and added additional auto-ionization transitions. Auto-ionization is the process whereby an electron in an excited state decays back to the ground state, releasing enough energy to spontaneously emit one of the outer shell electrons. Model DCA_79x3 included the new transitions from DCA_79x1 and also included doubly excited states up to n = 10, where n is the principle quantum number. Finally, DCA_79x5 started from DCA_79x3 and applied modifications to some computed auto-ionization rates to approximately match ENRICO. Figure 3 shows that as these changes were made to the DCA models, the computed emissivity for gold began to more closely approach the SCRAM curve. In particular, DCA_79x5 is within 20% of the SCRAM calculation over the full range of temperatures.

To assess the sensitivity of the calculated drive to the NLTE model, we calculated NIF shot N150506-002-999, a 5.75 mm diameter hohlraum filled with 0.85 mg/cc He gas, using either DCA_2010 or DCA_79x1. Since this model had the lowest emissivity of the new models we considered, it represents a maximum deviation from DCA_2010. Both calculations used a flux limiter of 0.15 and both included CBET. The CBET was calculated using the inline CBET package^23^ with the maximum δn/n set to 0.01. Because we were focused on the drive and bang time rather than the drive symmetry, the radiation drive was artificially symmetrized to force a symmetric implosion. We expect DCA_79x1 to be less effective in producing x-rays and, consequently, expect it to put more power into heating the coronal plasma.

We can approximately quantify the radiation production in a hohlraum simulation by post-processing to find

$$P_{xray}=P_{wall}+P_{r}+P_{resc},$$ (1)

where P_xray_ is the radiation production in the simulation, P_wall_ is the time derivative of the total energy in the simulated hohlraum wall (i.e., electron and ion internal and kinetic energy contained in zones with T_e_ < T_r_), P_resc_ is the radiation power escaping the simulation (almost all through the LEH), and P_r_ is the derivative of the radiation energy contained in the hohlraum (small).

We can then define the hohlraum x-ray conversion efficiency as

$$CE=\frac{P_{xray}}{\left(P_{las}-P_{lesc}\right)},$$ (2)

where P_las_ is the input laser power and P_lesc_ is the laser power that escapes the simulation (i.e., exits through an LEH).

**FIG. 4.**
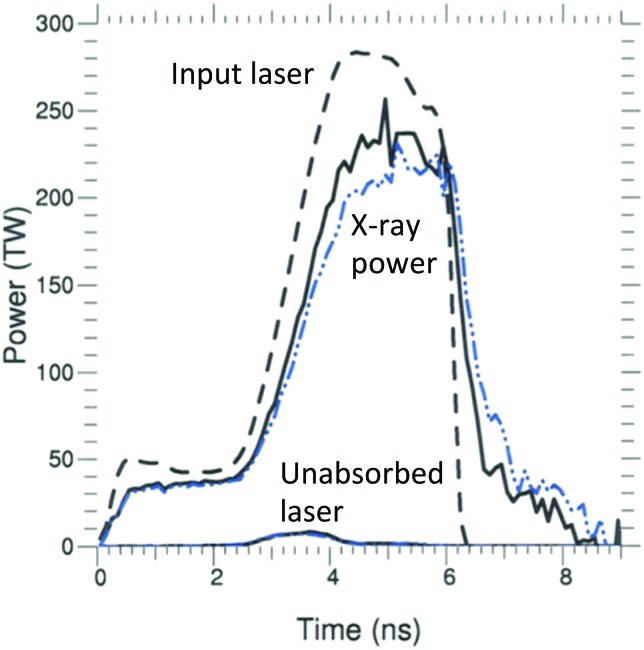
Plot of the input laser power (black dash), the unabsorbed laser power, and the net x-ray power produced for shot N150506-002-999. The solid black curves are for DCA_2010, and the dashed blue curves are for DCA_79x1.

**FIG. 5.**
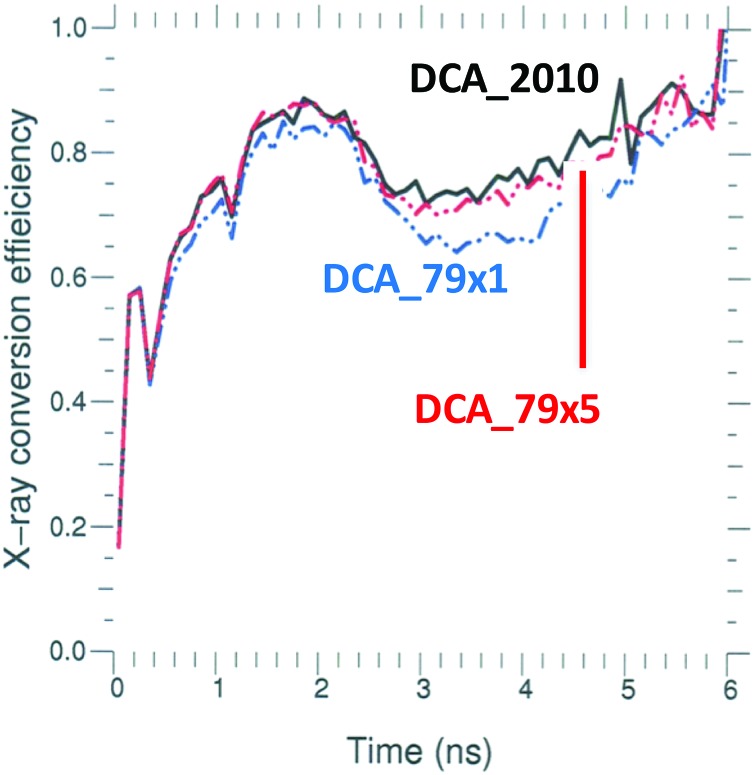
Calculated x-ray conversion efficiency for shot N150506-002-999 using DCA_2010 (solid), DCA_79x1 (blue dash), and DCA_79x5 (red dash).

Figure 4 shows that the calculation using DCA_79x1 does indeed produce about 10% less x-ray flux than the baseline model, DCA_2010. Also, note that during the increase in peak laser power, there is about 10 TW of laser power (mostly from the 23° beams) that is not absorbed and escapes out the opposite LEH with both NLTE models. Figure 5 shows that the x-ray conversion efficiency at peak power (∼5 ns) drops from ∼0.8 to ∼0.7 for DCA_79x1. For the most detailed DCA model, DCA_79x5, the difference is less, but the conversion efficiency is still 2%–3% lower than the original DCA_2010 model.

## III. EFFECT OF INHIBITED ELECTRON HEAT TRANSPORT

In flux-limited heat transport, the calculated heat flux is limited to a fraction, f, of the free-streaming heat flux in order to prevent unphysically high heat transport in regions where collisional mean free paths are not much shorter than T_e_ scale lengths. The HFM uses a flux limiter of 0.15. This value was based on the results of experiments on uniformly illuminated spheres.^11^ We hypothesize that in a laser-heated hohlraum, the heat transport could be inhibited relative to that inferred from uniformly illuminated sphere experiments. Possible sources of inhibition include non-local effects, laser-generated magnetic fields that arise from non-parallel density and temperature gradients,^24,25^ and plasma instabilities such as the two-stream instability that can create ion-acoustic turbulence.^26^

**FIG. 6.**
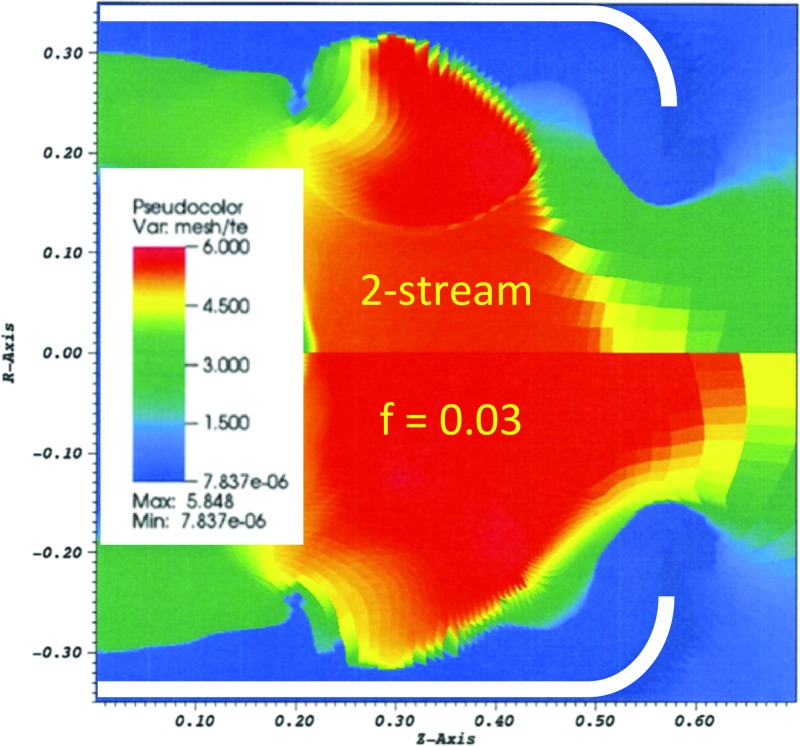
Electron temperature contours at 6 ns from calculations of N140701-002-999 using the two-stream model to inhibit heat transport (top half) or using a flux limiter of 0.03 (bottom half).

In the Spitzer-Harm formulation of heat flux, electrons with 2–4 times the thermal velocity carry most of the heat. To maintain zero net current, the bulk of the electrons drifts in the opposite direction relative to the ions, forming a cold return current. When this drift current exceeds the sound speed and the ion Landau damping is weak (ZT_e_/T_i_ ≫ 1), then the two-stream instability is triggered and can grow quickly into ion acoustic turbulence, which scatters energy and inhibits heat transport. In Lasnex, this effect can be included in a crude way by making the flux limiter a function of ZT_e_/T_i_ in each zone, where Z is the ionization state, T_e_ is the electron temperature, and T_i_ is the ion temperature. In the Lasnex two-stream model, the inverse of the flux limiter, 1/f, in each zone is specified as

$$f^{-1}=f_{o}^{-1}+\frac{\sqrt{\frac{ZT_{e}}{T_{i}}}}{1+\sqrt{\frac{ZT_{e}}{T_{i}}}\sqrt{\frac{Zm_{e}}{m_{i}}}},$$ (3)

where f_o_ is the user specified flux limit, which we took to be 0.15. In the limit where ZT_e_/T_i_ ≫ 1, the flux limiter becomes

$$f\approx \sqrt{\frac{Zm_{e}}{m_{i}}}=\frac{v_{s}}{v_{Te}},$$ (4)

where m_e_ is the electron mass, m_i_ is the ion mass, v_Te_ is the electron thermal velocity, and v_s_ is the ion sound speed. In this case, the heat flux, q, is then limited to

$$q=fn_{e}T_{e}v_{Te}=n_{e}T_{e}v_{s},$$ (5)

where n_e_ is the electron number density. For example, in gold zones with Z = 50 and ZT_e_/T_i_ = 200, this model reduces the flux limiter in those zones from 0.15 to 0.05. Note that this simple model only accounts for the damping and does not explicitly test whether the return current is unstable (drift speed exceeds sound speed). Figure 6 shows temperature contours at peak laser power (6 ns) for two calculations of N140701-002-999, a 6.72 mm diameter hohlraum filled with 0.6 mg/cc He gas. The top half is a calculation using this two-stream model. We see that the temperature in the gold is ∼6 keV. The bottom half of the figure uses a global flux limiter of 0.03 instead. This model is cruder but leads to a similar temperature in the gold plasma. Calculations using f = 0.15 have a peak electron temperature of ∼4 keV. For simplicity, in the following, we decided to use a global flux limiter of 0.03 as a surrogate model for heat inhibition.

**FIG. 7.**
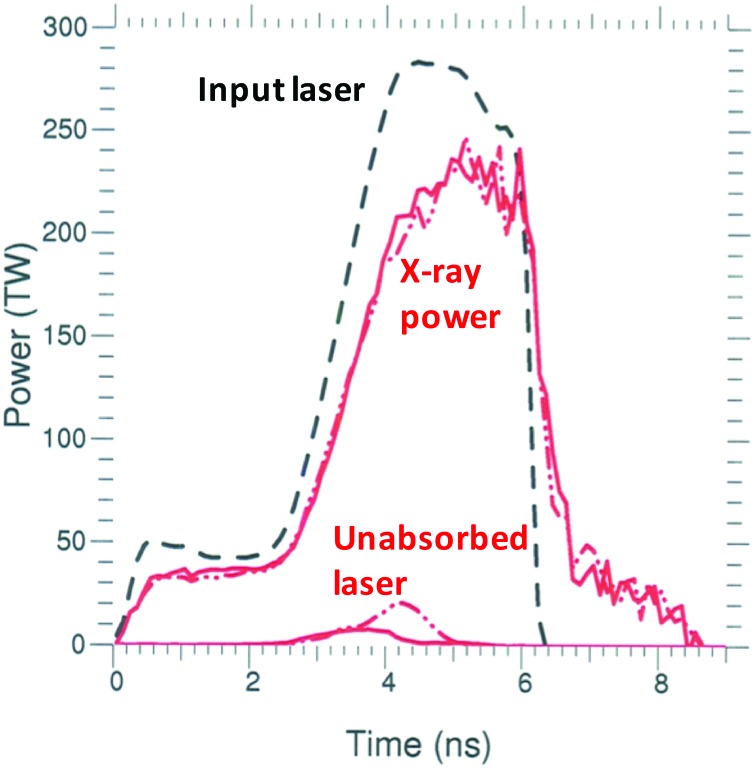
Plot of the input laser power (black dash), the unabsorbed laser power, and the net x-ray power produced for shot N150506-002-999. The solid red curves are for DCA_79x5 with f = 0.15, and the dashed red curves are for DCA_79x5 with f = 0.03.

**FIG. 8.**
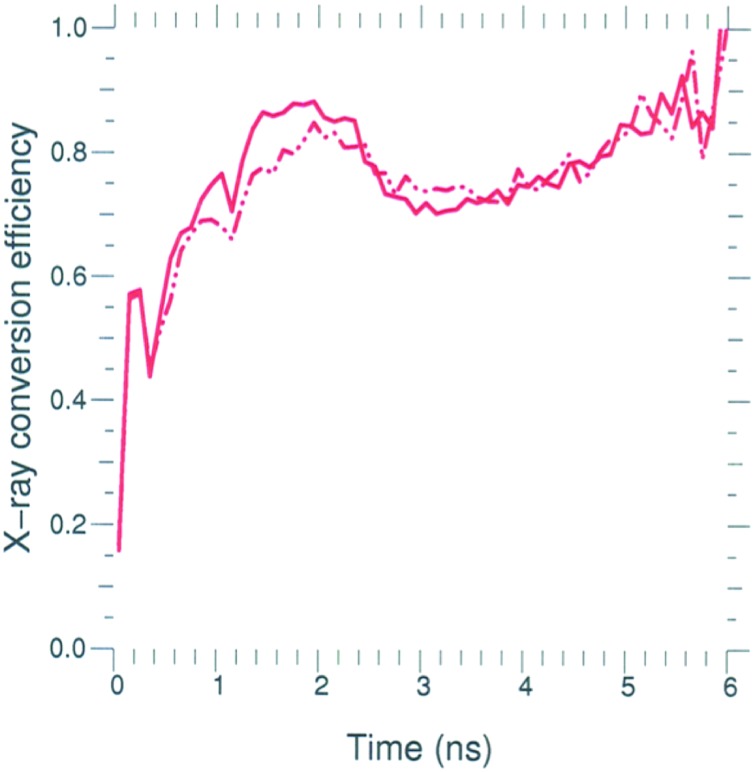
Calculated x-ray conversion efficiency for shot N150506-002-999 using f = 0.15 (solid) and f = 0.03 (dash).

The main effect of reducing the flux limiter from 0.15 to 0.03 is to increase the temperature of the plasma along the laser beam path and decrease the laser absorption. Figure 7 shows the x-ray power production and the unabsorbed laser power for calculations of N150506-002-999 using DCA_79x5 and f = 0.03 or f = 0.15. With a flux limiter of 0.15, the escaping laser power peaks at ∼10 TW during the increase in peak laser power. However, when the flux limiter is reduced to 0.03, the escaping power persists for a longer time and the peak loss rate is ∼20 TW. Almost all of the simulated escaping power is laser power in the 23° and 30° beams reflecting off the center of the cylindrical hohlraum and exiting the opposite LEH. We call this reflected energy escaping the opposite LEH “glint.” About 2/3 of the glint is in the 23° cone. Note that in contrast to modifying the NLTE model, we find that changing the flux limiter has very little effect on the hohlraum x-ray conversion efficiency during peak power, as shown in Figure 8. However, the gross radiation production is reduced with f = 0.03 because the hohlraum absorbs less laser energy.

There is experimental evidence that inner beam laser energy is glinting out the opposite LEH. However, the evidence is indirect because of the NIF geometry and diagnostic placement. NIF opposed beams are azimuthally “clocked,” and so, unabsorbed light from, say, a beam in the top hemisphere of NIF cannot glint through the opposite LEH and go directly into a beam port on the bottom hemisphere. The current diagnostic that potentially could quantify glint [Full Aperture Backscatter Station (FABS)/Near Backscatter Imager (NBI)] is mounted on a 30° beam port (Q31B) in the lower hemisphere. Because it is “clocked” by ∼22.5° from where the center of glint from an upper beam (e.g., Q33T) would strike the bottom hemisphere of the chamber, it will only detect the wings of any glint.

**FIG. 9.**
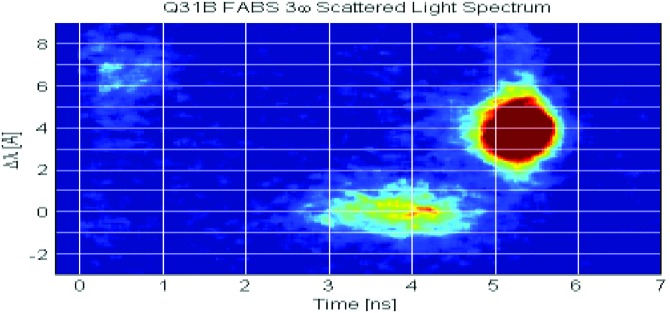
SBS data for shot N150228-002-999. Blue-shifted light appearing on the SBS spectrometer between 3 and 4 ns is attributed to unabsorbed laser light from the opposite 23° beams and is easily distinguished from the red-shifted backscatter occurring at the time of peak power.

A spectrometer in Q31B designed to measure the stimulated Brillouin backscatter (SBS) into the lens provides the indirect evidence for glint and its timing. It has previously been used by Turnbull *et al*.^27^ to infer glint losses in a NIF vacuum hohlraum. Figure 9 shows the SBS spectrometer data from shot N150228-002-999, another 2-shock HDC capsule in a 5.75 mm diameter hohlraum, but with a 1.6 mg/cc He hohlraum fill. The slightly blue-shifted signal appearing between 3 and 4 ns, which is the time of the rise to peak laser power, can be attributed to glint from Q33T. The slight blue shift is what we would expect from a reflection off a hohlraum wall that moves radially. The later SBS signal, red shifted by ∼4 A, is the signature of the true SBS backscatter. The slightly blue shifted measurement is not quantitative because the main glint would be striking the NIF chamber wall some 22.5° away. We note that the situation is further complicated by the fact that the unabsorbed light escaping the LEH can undergo CBET with light from other beams entering the LEH.^27^

## IV. APPLYING A NEW MODEL TO SEVERAL GAS-FILLED HDC EXPERIMENTS

**FIG. 10.**
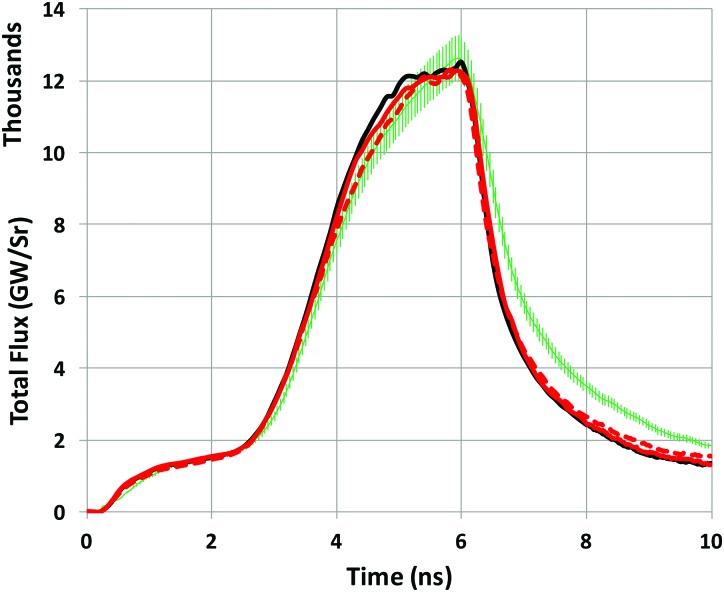
Measured total Dante signal (green with error bars) for shot N150506-002-999 compared with the simulated Dante signal from calculations with DCA_2010 and f = 0.15 (black), DCA_79x5 and f = 0.15 (red), and DCA_79x5 and f = 0.03 (red dash).

**FIG. 11.**
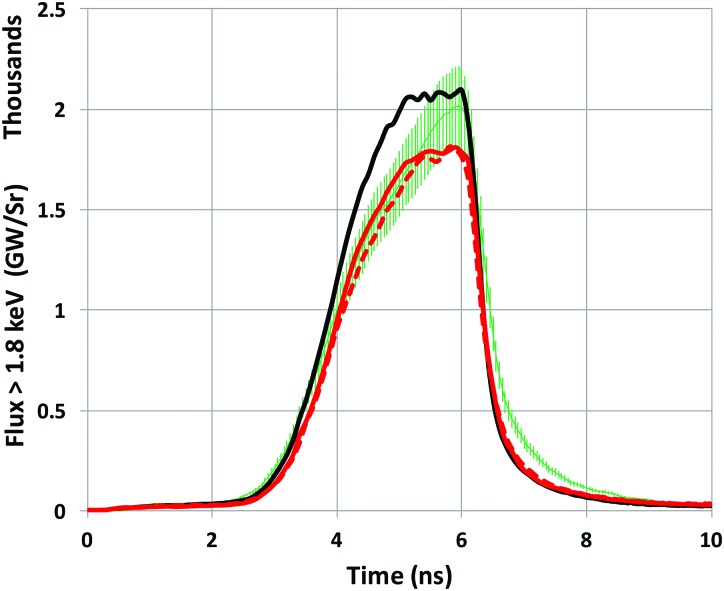
Plots of the “M-band flux” (flux > 1.8 keV) versus time. The measured M-band flux (green with error bars) for shot N150506-002-999 is compared with the simulated Dante M-band signal from calculations with DCA_2010 and f = 0.15 (black), DCA_79x5 and f = 0.15 (red), and DCA_79x5 and f = 0.03 (red dash).

**FIG. 12.**
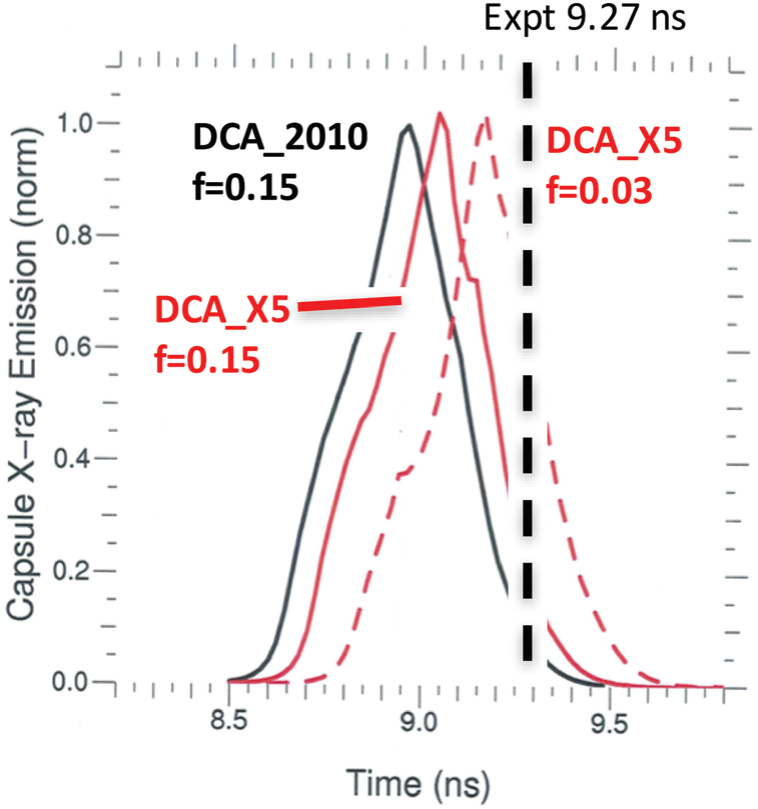
Measured capsule x-ray bang time for shot N150506-002-999 compared to simulated x-ray emission curves from calculations with DCA_2010 and f = 0.15 (black), DCA_79x5 and f = 0.15 (red), and DCA_79x5 and f = 0.03 (red dash).

A model combining a fully converged grid (space, time, and energy), DCA_79x5 (which has improved agreement with detailed SCRAM calculations), and a flux limiter of f = 0.03 leads to better agreement with radiation drive data without requiring ad hoc adjustments to the input laser power. The data we use to infer the radiation drive are the spectrally resolved time-dependent flux measured out of the LEH by the Dante diode array^28^ and capsule bang time. The Dante data unfold procedure produces a spectrally resolved radiation flux at each time point. By integrating the spectrum across the full energy range of the diode array (out to 13 keV), the total spectrally integrated flux as a function of time is obtained. Figure 10 compares the total experimentally measured Dante flux for N150506-002-999 to the simulated Dante flux extracted from a calculation using the original HFM (black curve), a calculation using DCA_79x5 (red curve), and one using DCA_79x5 and f = 0.03 (red dashed curve). The original HFM is higher than the data, especially during the increase in peak power. The modifications bring the simulations closer to the data. The difference between the new and old models is mostly in the high-energy part of the spectrum that comes directly from the laser-heated spots. To illustrate this, Figure 11 shows the measured and calculated fluxes for photon energies >1.8 keV, which highlights the non-Planckian gold M-band portion of the spectrum. Changing to the new DCA_79x5 model brings the M-band flux down to within the 10% measurement error bars during the increase in the pulse. The flux limiter has only a minor effect on the M-band flux. This result is consistent with Figure 3, which showed that the DCA_2010's largest deviation from the SCRAM emissivity was for photon energies above 2 keV. A discrepancy remains after the laser shuts off, with the measured Dante signals consistently higher than all the simulations. Figure 12 shows the calculated x-ray bang time compared to the measured x-ray bang time for the same three simulations. We see that the two model modifications delay the calculated x-ray bang time by over 200 ps relative to the original HFM calculation, bringing the bang time to within 100 ps of the measured value.

Since the f = 0.03 model predicts a hotter plasma in the laser channel, a natural question is whether there are data to support this. Recently, a technique to measure the plasma temperature using a spectroscopic technique has been developed.^29^ In this technique, a “microdot” (3200 A thick) of a 50/50 mixture of Mn and Co is placed on the pole of a plastic capsule in the hohlraum. As the tiny dot of the Mn/Co material moves into the laser-heated part of the plasma, it begins to emit and the time-dependent spectrum is measured by using a spectrometer looking at the material through the LEH. The measured line ratios are used along with SCRAM calculations to determine the temperature of the dot material. NIF shot N160424-001-999 was a microdot experiment in a 5.75-mm diameter hohlraum filled with 0.6 mg/cc He. This experiment was unique in several ways. All previous microdot experiments had been done in “viewfactor hohlraums,” which are open at one end and have a thin (20 *μ*m) shell that is designed to become transparent to x-rays by the time of peak power to allow an unobstructed view of the hohlraum wall. Viewfactor shells do not provide a bang time measurement. However, shot N160424-001-999 was done in standard two-sided hohlraum. This experiment has the same hohlraum dimensions, gas fill, and laser pulse shape as the HDC experiment (N150301-003-999). In addition, the microdot was placed on the pole of a standard 190 *μ*m thick CH symmetry capsule. The capsule was filled with the same gas as the hohlraum (0.6 mg/cc He4). This unique arrangement allowed us to simultaneously measure the plasma temperature and x-ray bang time on the same shot. This experiment was essentially identical to N150301-003-999, except with the HDC capsule replaced with a CH capsule.

**FIG. 13.**
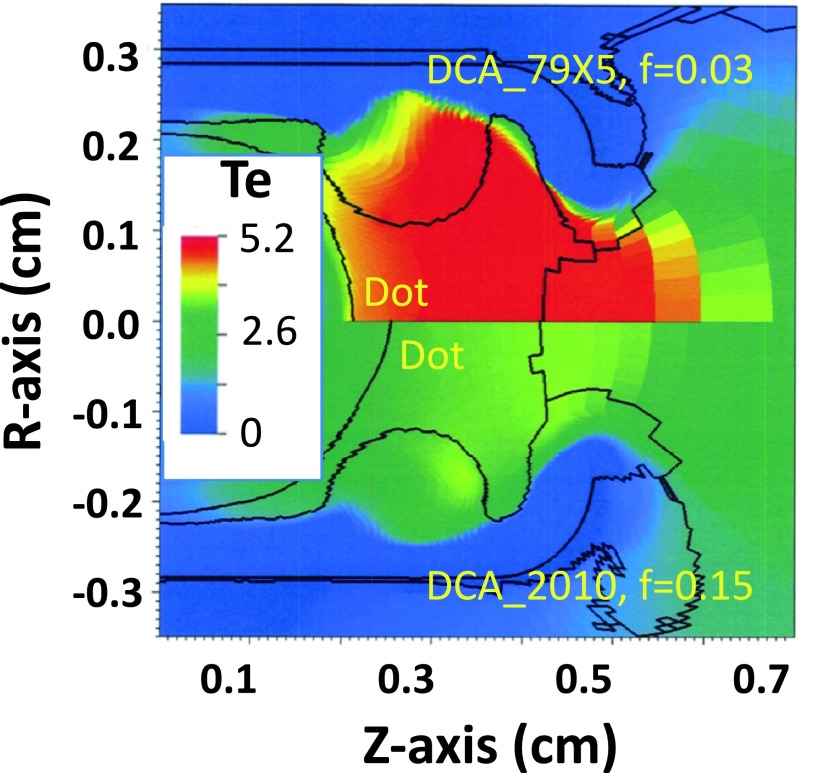
Electron temperature and material boundary positions at 5.2 ns for the N160424-001-999 0.6 mg/cc He fill dot spectroscopy experiment. The top half is for DCA_79x5 and f = 0.03, while the bottom is for DCA_2010 and f = 0.15.

**FIG. 14.**
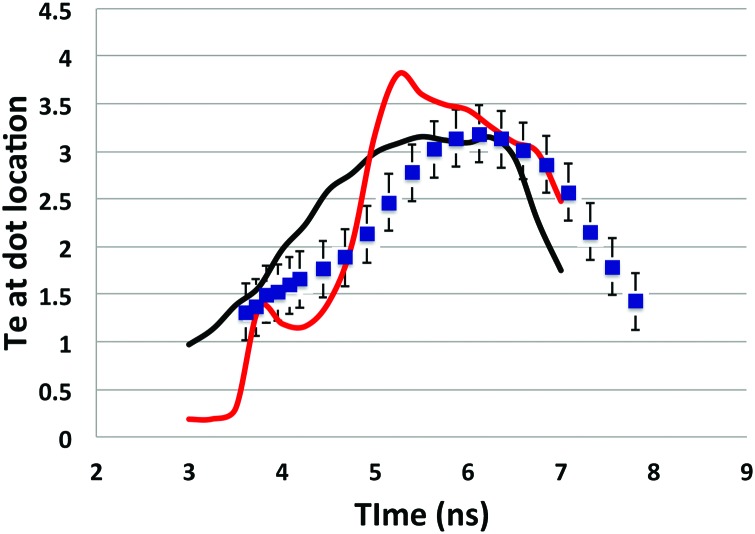
Measured dot temperature vs. time from N160424-001-999 with measurement uncertainty (blue squares) compared to the dot temperature for two simulations. The black curve is for DCA_2010 and f = 0.15. The red curve is for DCA_79x5 and f = 0.03.

Figure 13 shows T contours at peak power (5 ns) for 2 simulations of N160424. The top half uses the new model with DCA_79x5 and f = 0.03, while the bottom half uses the old HFM (DCA_2010, f = 0.15). The new model is ∼2 keV hotter in the LEH, but as you get closer to the location of the dot material, which is well inside the LEH, the temperature difference between the two simulations is much smaller. Also, note that the f = 0.03 model results in a large temperature gradient just outside the dot location and that the higher temperature outside the capsule causes a higher pressure that slows the expansion of the capsule relative to the HFM calculation. Barrios *et al*.^29^ found in their viewfactor experiments that HFM calculations generally under-predicted the plasma temperature by about 0.5 keV and over-predicted the expansion rate of the capsule. A more restrictive heat flux does tend to move the calculations in a direction to compensate for those discrepancies. Figure 14 shows the temperature of the dot material from N160424 and compares it to the calculated temperature at the dot position for the two simulations of N160424. We see that neither model agrees with the dot temperature data for all times. Both models disagree with the temperature data up to about 5.5 ns. The original HFM with f = 0.15 comes into agreement during late peak power, but the temperature drops faster than the data after the laser turns off. The alternate f = 0.03 model predicts too high a temperature early in the peak but comes into agreement with the data at the end of peak power and after the laser turns off. Because the procedure for extracting the temperature for the dot material line ratios assumes the steady state, the technique could tend to underestimate the temperature during the main rise (2–3 ns) and overestimate the temperature when the laser shuts off and the plasma cools (6–7 ns). It is therefore possible that some of the HFM model discrepancy during the rising and falling parts of the laser pulse could be explained by this effect. Although it appears that using a global flux limiter of 0.03 is too crude for a model to explain the details of the plasma temperature evolution, the dot spectroscopy temperature data do not rule out the hypothesis of some sort of inhibited heat transport, especially late in time when the data suggest that the plasma cools more slowly than predicted using f = 0.15.

**FIG. 15.**
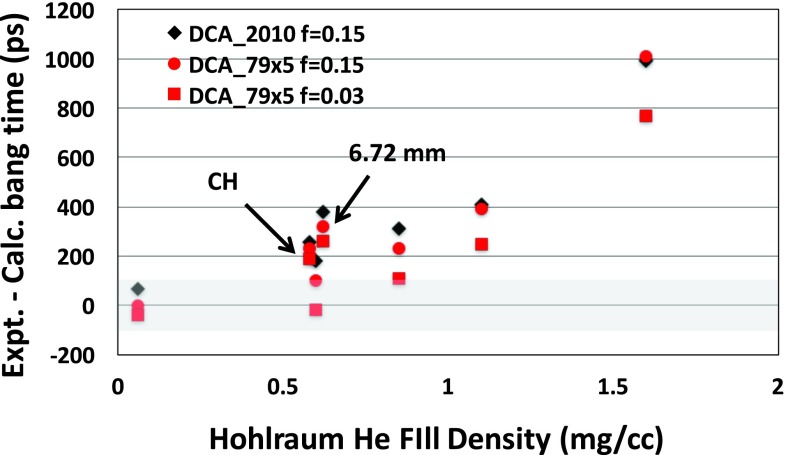
Difference between the experiment and calculated x-ray bang times for 2-shock implosions in 5.75 mm and 6.72 mm diameter hohlraums over a range of He hohlraum fill gas densities using DCA_2010 and f = 0.15 (black diamonds), DCA_x5 and f = 0.15 (red circles), and DCA_x5 and f = 0.03 (red squares).

Finally, the new model was applied to all the experiments listed in Table I. The data set includes experiments in two hohlraum sizes (5.75 and 6.72 mm diameter) and spanned a range of hohlraum fill densities from 0.06 to 1.6 mg/cc He. Figure 15 shows a plot of the difference between the experimentally measured x-ray bang time and the calculated x-ray bang time. A positive value means that the calculated x-ray bang time is earlier than the data. Given that the laser energy is known to about 3% accuracy and that for these pulses a 1% uncertainty in the radiation flux results in ∼30 ps in bang time uncertainty, we estimate that predicting the bang time to within ±100 ps would be within our measurement uncertainties. The gray band in Figure 15 shows this estimated uncertainty. The black diamonds are using the original HFM without any adjustments. We see that the calculated bang times are generally early relative to the data and that the discrepancy becomes bigger for higher gas fill densities. The red circles are the calculations with DCA_79x5 and f = 0.15, and the red squares are using the new model with DCA_79x5 and f = 0.03. This new model predicts the bang time to within 100 ps for 3 of the 5 shots with fill densities of 0.85 mg/cc or less without using any ad hoc multipliers on the input laser power. The new model delays the bang time by about 200 ps for the higher fill experiments as well, but those calculated bang times are still much earlier than measured. For the lowest fill, the new DCA model had a bigger impact than the flux limiter change. At the intermediate fills, both changes had comparable effects. At the two highest fill densities, the flux limiter change is dominant. This model predicts a slight increase in the amount of laser energy glinting and escaping the opposite LEH as the fill density is increased. The reason for this is that as the density is increased, hot gold in the laser path is replaced by hot He, which absorbs less and lets more light escape. This prediction could be experimentally tested. Notice that there is some scatter in the results. For example, the calculations at a fill density of 0.6 mg/cc for the larger 6.72 mm hohlraum and for the CH shell have relatively larger x-ray bang time discrepancies than for the comparable point with HDC in the 5.75 mm hohlraum. All these calculations did have some measured backscatter removed from the input laser power (see Table I), and so, uncertainties in the backscatter measurement could be one cause of the scatter.

## V. CONCLUSIONS

In this work, we introduce a new model that brings the calculated bang time into better agreement with the measured bang time for experiments with low to intermediate hohlraum fill densities. The new model employs (1) a fine grid that is fully converged in space, energy, and time, (2) a modified approximate NLTE model that includes more physics and is in better agreement with more detailed offline emissivity models, and (3) a reduced flux limiter value of 0.03. We applied this model to gas-filled hohlraum experiments using high density carbon ablator and plastic capsules that had hohlraum He fill gas densities ranging from 0.06 to 1.6 mg/cc and hohlraum diameters of 5.75 or 6.72 mm. The model can predict bang times to within ±100 ps for most experiments with low to intermediate fill densities (up to 0.85 mg/cc) without requiring ad hoc adjustments to the input laser power. This model predicts higher temperatures in the plasma than the old model and also predicts that at higher gas fill densities, a significant amount of laser energy can escape the hohlraum through the opposite laser entrance hole. The main effect of the modified NLTE model is to slightly lower the peak x-ray conversion efficiency, whereas the main effect of restricting the heat transport via the reduced flux limiter is to make the plasma hotter and less absorbing along the inner beam path, resulting in the light escaping out the opposite LEH.
